# Assessing Configurational Sampling in the Quantum Mechanics/Molecular Mechanics Calculation of Temoporfin Absorption Spectrum and Triplet Density of States

**DOI:** 10.3390/molecules23112932

**Published:** 2018-11-09

**Authors:** Martina De Vetta, Omar Baig, Dorika Steen, Juan J. Nogueira, Leticia González

**Affiliations:** 1 Institute of Theoretical Chemistry, Faculty of Chemistry, University of Vienna, Währinger Str. 17, 1090 Vienna, Austria; martina.de.vetta@univie.ac.at (M.D.V.); a01200376@unet.univie.ac.at (O.B.); 2 Departamento de Química Universidad Autónoma de Madrid Francisco Tomas y Valiente, 7, Cantoblanco, 28049 Madrid, Spain; 3 Biolitec Research GmbH, Otto-Schott-Str. 15, 07745 Jena, Germany; Dorika.Steen@biolitec.com

**Keywords:** Temoporfin, configurational sampling, QM/MM, absorption properties

## Abstract

The absorption properties of Temoporfin, a second-generation photosensitizer employed in photodynamic therapy, are calculated with an electrostatic-embedding quantum mechanics/molecular mechanics (QM/MM) scheme in methanol. The suitability of several ensembles of geometries generated by different sampling techniques, namely classical-molecular-dynamics (MD) and QM/MM-MD thermal sampling, Wigner quantum sampling and a hybrid protocol, which combines the thermal and quantum approaches, is assessed. It is found that a QM description of the chromophore during the sampling is needed in order to achieve a good agreement with respect to the experimental spectrum. Such a good agreement is obtained with both QM/MM-MD and Wigner samplings, demonstrating that a proper description of the anharmonic motions of the chromophore is not relevant in the computation of the absorption properties. In addition, it is also found that solvent organization is a rather fast process and a long sampling is not required. Finally, it is also demonstrated that the same exchange-correlation functional should be employed in the sampling and in the computation of the excited states properties to avoid unphysical triplet states with relative energies close or below 0 eV.

## 1. Introduction

**Scheme 1:**
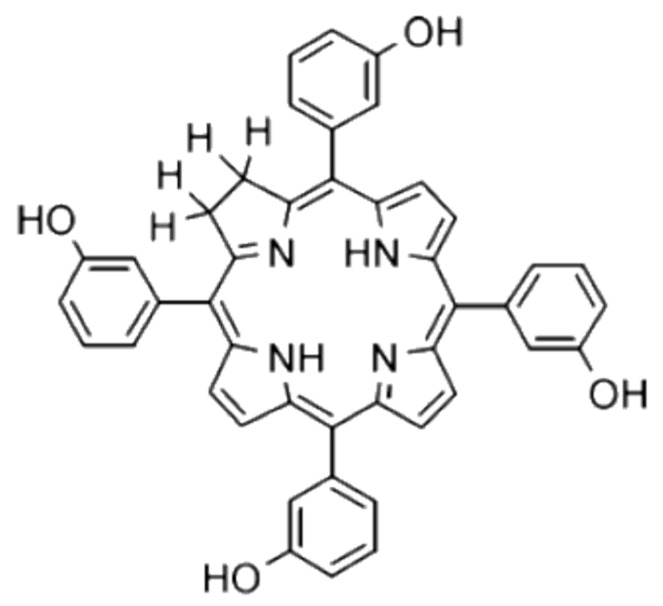
Structural representation of the second-generation photosensitizer mTHPC (5,10,15,20-tetra(m-hydroxyphenyl)chlorin).

Photodynamic therapy—an established treatment for certain types of cancer [1,2,3,4]—is nowadays also employed against infectious diseases, dermatological conditions and diabetes [5,6,7,8]. This therapy exploits the excited-state reaction of a photosensitive compound, called photosensitizer (PS), with molecular oxygen and/or target biomolecules of the cells, after being irradiated with light, resulting in singlet oxygen or other cell-toxic products [4,9,10]. Currently, porphyrins and derivatives dominate the types of compounds employed as PSs in therapeutic use. In fact, the first PS ever used commercially in photodynamic therapy was a mixture of hematoporphyrin derivatives, currently contained in the drug Photofrin [11,12,13]. This PS was far from ideal as it consisted of a mixture of mono- and oligomers, that is, a single active compound could not be isolated. Thus, the second generation of PSs aimed at a singleunique photoactive phorphyrinoid compound [14,15]. One class of compounds that resulted from this quest is hydroporphyrins, such as the meso-tetra(hydroxyphenyl)chlorins. These compounds were designed to allow the solubility in polar media by the introduction of hydroxyl groups and to enhance light absorption in the lower-energy part of the spectrum [14]. It is in fact important to have intense light absorption in the so-called therapeutic window (630–850 nm), where most of the tissue components are quite transparent and the radiation can penetrate more in depth [16]. This can be achieved by reducing porphyrins to their correspondent chlorins, for which the intensity of the most red-shifted absorption Q-band is enhanced by disruption of the structural symmetry [14,16,17]. The species 5,10,15,20-tetra(m-hydroxyphenyl)chlorin (Temoporfin or mTHPC), shown in Scheme 1, belongs to this series of reduced porphyrins and has been successfully developed into a photodrug known with the proprietary name of Foscan^®^ [18,19]. mTHPC is currently employed in clinics [1,2,3,13,19,20,21] and it is being further developed into an assisted delivery formulation, where the drug is embedded in liposomes [22]. This new formulation should expand the applications of the drug and improve its efficacy [23,24,25,26].

The absorption and photodynamical properties of mTHPC have been also studied computationally by means of time-dependent density-functional theory (TD-DFT) calculations [17,27,28,29], where solvent effects were sometimes included by implicit models [17,28]. In implicit solvation models the whole environment is treated as a polarizable continuum [30] that represents the macroscopic characteristics of the solvent [31]. Such a simplified description is satisfactory for the investigation of many solvated chromophores, even when explicit interactions, such as hydrogen bonds, are present [32]. In other cases, however, the introduction of explicit solvent molecules and solvent sampling is mandatory because an average description of the environment is not able to properly describe the solvent effects or because the environment has a heterogeneous structure, for example, as in the case of biological environments, such as proteins, DNA or lipid membranes [28].

When the environment is included explicitly, a popular approach is to divide the system into several regions that are described at different levels of theory with different accuracy [33,34]. Although more than one layer is possible [35], very commonly, the system is split into two regions: one containing the chromophore, which is treated accurately with quantum mechanics (QM) and another one describing the environment at a lower level of theory, often at the molecular mechanics (MM) level by means of force fields. The use of these so-called QM/MM schemes is less extended than QM/continuum ones because the introduction of explicit solvent in the former is more laborious [36].

In some cases, relevant molecules that belong to the environment have to be included in the QM region with the chromophore in order to describe, for example, charge-transfer states between the solute and environment or to correctly describe solute-solvent polarization effects [37]. However, the definition of a correct and large enough QM region plays a very important role in the accuracy of the computed property and is often not straightforward to define [38]. Polarization between solute and solvent can also be accounted by means of a polarizable-embedding QM/MM scheme, which oftentimes even allows to reduce the size of the QM region to the chromophore alone [39].

When the solvent molecules are represented explicitly, many solvent organizations around the chromophore are possible. Therefore, sampling of the configurational space prior the computation of the desired property is compulsory [36,40]. Two approaches are generally employed for the configurational-space sampling, namely, thermal sampling and quantum sampling. In the first case, a ground-state molecular-dynamics (MD) simulation is performed, in which the system is in thermal equilibrium at a certain temperature. In the quantum sampling, the configurational space is represented by the quantum-mechanical distribution of the population of the vibrational ground state if one assumes a temperature of 0 K; if temperature effects are included, the distribution also considers the population of vibrationally excited states of the electronic ground state [41]. Both approaches can also be combined by performing quantum sampling for the chromophore and thermal sampling for the solvent. It is important to note that the inclusion of vibrational sampling properly accounts for the different chromophore geometries, interactions between chromophore and solvent as well as temperature effects, which are key to determine the shape of the spectrum. However, vibronic transitions, whose description requires the computation of the vibrational wavefunctions of the initial and final states, are still neglected in these sampling approaches. Few formulations accounting for vibronic transitions in an ensemble of geometries have been developed [37]. However, the use of these vibronic approaches is beyond the scope of the present work, whose aim is to assess the performance of different vibrational sampling techniques.

In this work, we have computed the absorption spectrum and the density of triplet states of mTHPC in methanol based on several ensembles of geometries. The ensembles were generated by thermal sampling based on classical and QM/MM MD, quantum sampling and a combination of both, named hybrid-Wigner-MD distribution. The quality of the different sampling approaches was assessed by comparison with the experimentally measured energies of the most-red shifted Q-bands, being the low-energy one employed for photodynamic therapy, and the most intense Soret band.

## 2. Results and Discussion

### 2.1. Absorption Spectrum

The study of biologically or biomedically relevant molecules is tightly linked to their interaction with the surrounding medium. A correct sampling of the geometrical configurations of the chromophore and solvent molecules and of the interactions between them is thus of fundamental importance for the computation of the properties of the chromophore. Once the ensemble of geometries has been created with the chosen sampling method, the properties of the chromophore can be computed for each geometry. This procedure delivers a probability distribution for each molecular property. In the case of the absorption spectrum, a probability distribution of the excitation energies of the different electronic states, weighted by their oscillator strengths, is obtained. A straightforward approach to sample the geometrical configurations is to perform a classical MD simulation of the chromophore of interest in the desired environment. From this simulation, a set of uncorrelated snapshots are extracted, providing a series of Boltzmann-distributed geometries of the chromophore and solvent. The geometries provided with the selected snapshots are used for the subsequent QM/MM calculation employing, usually, an electrostatic embedding scheme [42,43,44,45] to compute the excitation energies. When the solvent molecules do not actively participate in the excitation process only the chromophore is usually included in the QM region.

**Figure 1:**
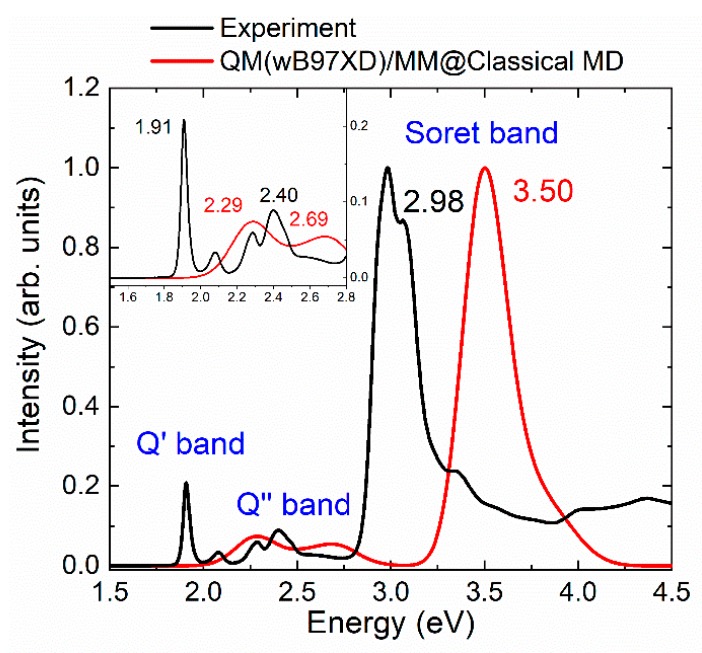
Comparison of the QM(wB97XD)/MM@Classical-MD spectrum (red curve) and the experimental spectrum (black), scaled with respect to the Soret band. In the inset a zoom on the Q-bands with the corresponding energy values at the maxima in eV is reported.

As first step, the combination of classical MD for sampling and electrostatic-embedding QM/MM for excitation energies has been employed to calculate the absorption spectrum of mTHPC in methanol (Figure 1). The QM region comprises the chromophore, for which the first six lowest-energy excited singlet states were computed at TD-wB97XD level of theory. This approach is named here QM(wB97XD)/MM@Classical-MD. The selection of the functional was based on a benchmark study performed at the ground-state optimized geometry and considering vibrational sampling, see Sections S1 and S2 of the Supplementary Materials. From the calculations, we deem the wB97XD functional the one providing the most accurate spectrum (in terms of energies and intensities) in the low-energy region, which is the relevant region for PDT, in terms of energies and intensities.

The quality of the theoretical approach has been evaluated by considering the error in the computed absorption spectrum with respect to the experimental one. Specifically, the low-energy part of the spectrum, which includes the Q′ and Q′′ bands (see inset of Figure 1) and the more intense Soret band (Figure 1) have been analyzed. As we can see, the predicted spectrum presents an error of 0.52 eV for the Soret band. This error is reduced but not yet acceptable, for the Q bands: 0.38 eV for the most red-shifted Q’ band and 0.29 eV for the Q′′ band.

Since the QM(wB97XD)/MM@Classical-MD absorption spectrum significantly differs from the experimental one, the effect of different sampling approaches will be investigated to find the source of the error. From now on, we will discuss the results of the different sampling approaches that generate the ensemble of geometries from which the spectrum is computed. In the nomenclature for the corresponding theoretical spectra, for example, QM(wB97XD)/MM@Classical-MD, we will omit the level of theory for the excited-state calculations, as it is the same throughout this first section (unless stated otherwise). Thus, we will identify the different spectra only from the sampling technique.

The large discrepancy between the experimental and the theoretical absorption spectra obtained with classical MD followed by QM/MM excited-state calculations (Figure 1) could be attributed to a failure of the force field that describes the vibrational motion of the chromophore during the classical sampling [46]. To investigate whether this is the case, the classical MD sampling is refined with subsequent QM/MM-MD simulations, where mTHPC is treated at QM level with the B3LYP functional. To perform the refined sampling, we selected 100 snapshots from the last nanosecond of the classical MD simulation and used them as starting points for 100 QM(B3LYP)/MM-MD simulations during ca. 100 fs each. Then, the last snapshot of the 100 short simulations is selected to form a new ensemble of geometries to compute the absorption spectrum of mTHPC. The selection of 100 snapshots from a relatively long interval (1 ns) from the classical MD trajectory ensures a good sampling for the solvent molecules, which often need a relatively long sampling to describe the diffusion of the solvent around the chromophore. In addition, the QM treatment of the chromophore along the short QM/MM-MD simulations is expected to improve the description of its vibrational motion, which needs a shorter sampling than the solvent.

One important question in this sampling approach is the choice of the QM method for the MD simulations. In our case, the selected level of theory was B3LYP/6-31G*, which is rather modest in comparison to the protocol designed for the excited-state calculations (wB97XD/6-311G**). Using a lower level of theory for the QM/MM-MD simulation to sample the configurational space seems a reasonable approach due to the large number of gradient calculations required for the MD trajectories. Moreover, the use of a lower-level method to compute the geometry and a higher-level method to compute the properties is also a common practice in static excited-state calculations, where the vertical-excitation energies are computed at the optimized ground-state geometry. This scheme is in principle valid because ground-state properties are often very well described by the golden standard B3LYP/6-31G*, whereas more sophisticated approximations of the exchange-correlation functionals are usually required for the computation of excited-state properties [47,48,49]. Lastly, the use of simple functionals and small basis sets for the sampling or the geometry optimization reduces the computational cost considerably, often without compromising the accuracy of the final results.

**Figure 2:**
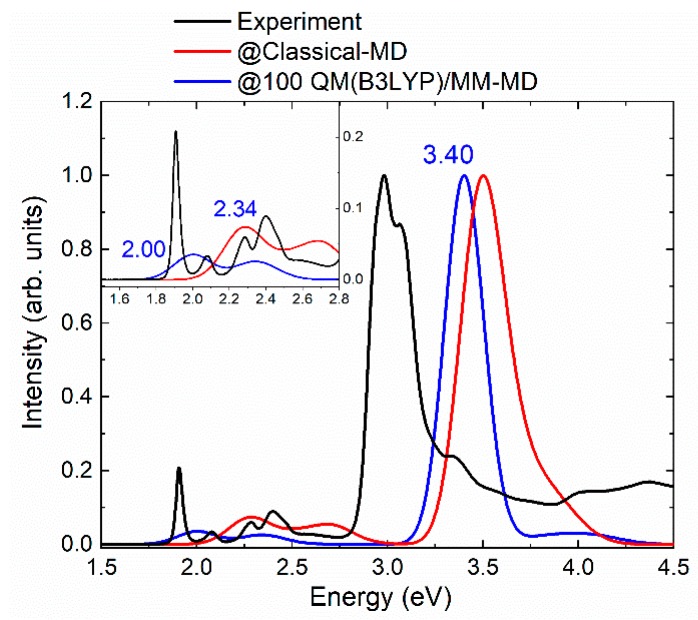
Comparison of the @100-QM(B3LYP)/MM-MD spectrum (blue) with respect to the experimental absorption curve of mTHPC in methanol (black) and the one obtained with the classical MD sampling (red). The spectra are scaled with respect to the Soret band. The inset zooms the Q’ and Q′′ bands. Values of the three peaks of the @100-QM(B3LYP)/MM-MD spectrum are given in eV.

Figure 2 displays (blue line) the absorption spectrum computed with the, then labelled @100-QM(B3LYP)/MM-MD ensemble of geometries. For the Soret band, which peaks at 3.40 eV, an improvement of 0.10 eV is achieved with respect to the classical MD-based Soret band (in red). However, the Q-bands (see inset) profited most from the benefits of the QM/MM-MD sampling. The lower lying Q’ band appears now at 2.00 eV, almost 0.3 eV lower in energy than the band predicted with the classical MD sampling. The higher-energy Q-band (Q’’ band) is found at 2.34 eV, red-shifted with respect to the experimental value by only 0.06 eV, so that it significantly improved the value of 2.69 eV obtained by the classical MD sampling approach. 

Although the error of the computed absorption spectrum is significantly reduced when the @100-QM(B3LYP)/MM-MD protocol is employed for sampling, there is still room for improvement. One thinkable issue could be the electrostatic-embedding scheme adopted for the excited states calculations. Methanol is a rather polarizable solvent for which more sophisticated QM/MM schemes, for example, a polarizable embedding, might provide more reliable results. In order to explore the plausible error due to missing polarization effects, we have recomputed the spectrum including 12 methanol molecules in the QM region during the electrostatic-embedding QM/MM vertical-energy calculations. In fact, it is sometimes important to include the most relevant regions of the environment in the QM region in QM/MM calculations or switch to more advanced techniques, where solvent polarization effects are included [39]. The computational details of these calculations are reported in Section S3 of the Supplementary Materials. As can be seen in Figure S4, the QM/MM calculation which describes solvent molecules at QM level provides virtually the same spectrum as the standard QM/MM calculations in which all the methanol molecules are described by a force field in the MM region. Therefore, one can conclude that, for this particular system, the polarization of the solvent does not affect the calculated absorption properties of the chromophore and, thus, the use of an electrostatic-embedding QM/MM scheme is appropriate.

The improvement obtained by describing the PS quantum mechanically during the configurational sampling can have two origins: (i) the general shape of the gradient of the PS was badly approximated by the force field adopted in the classical MD simulation leading to wrong geometries, or (ii) there are strong anharmonicities in the chromophore motion which are not captured by the harmonic force field.

Standard force fields can in principle account for a small degree of anharmonicity, even when bonds and angles are described by harmonic potentials, due to the non-bonded terms, that is, coulombic and van der Waals interactions, which describe long-range interactions between non-connected atoms. Nevertheless, one can say that the vibrational motion provided by standard force fields is mainly harmonic and the use of QM/MM-MD for sampling describes the anharmonicities much better. To distinguish between these two possible factors affecting the quality of the calculated absorption spectrum, we have also employed an ensemble of geometries obtained from a Wigner distribution (quantum sampling), where each normal mode is described with a quantum-mechanical harmonic oscillator. Each degree of freedom has its zero-point energy, which is normally higher than the thermal energy *k_b_T* provided by the sampling approaches based on classical or QM/MM MD. A configurational space generated with quantum sampling is therefore generally hotter than the correspondent ensemble generated with a thermal sampling. Only the low-frequency modes could receive the same amount of energy in both sampling approaches and those are also the modes that could deviate most from the harmonic approximation. As the 100 geometries obtained with the quantum sampling are strictly harmonic, the comparison with the QM/MM-MD approach allows determining whether the improvement in the QM/MM-MD-based absorption spectrum with respect to the classical MD-based one is due to a better description of the anharmonic motions or not. In order to keep the comparison rigorously bound to the harmonic character of the configurations of the chromophore in the ensemble, the solvent has to be described in the same fashion as in the QM/MM-MD ensemble. This was achieved by taking the previously selected 100 snapshots of the classical MD simulation and replacing the geometries of the chromophore by the geometries from the Wigner distribution. Such snapshots were then the starting point for 100 classical simulations of 1 ns, where the geometry of the drug was kept frozen and the solvent relaxes to adapt to the new structure of the chromophore. The hybrid-Wigner-MD ensemble formed by the last snapshot from each of these 100 classical MD simulations was employed for excited-state calculations.

**Figure 3:**
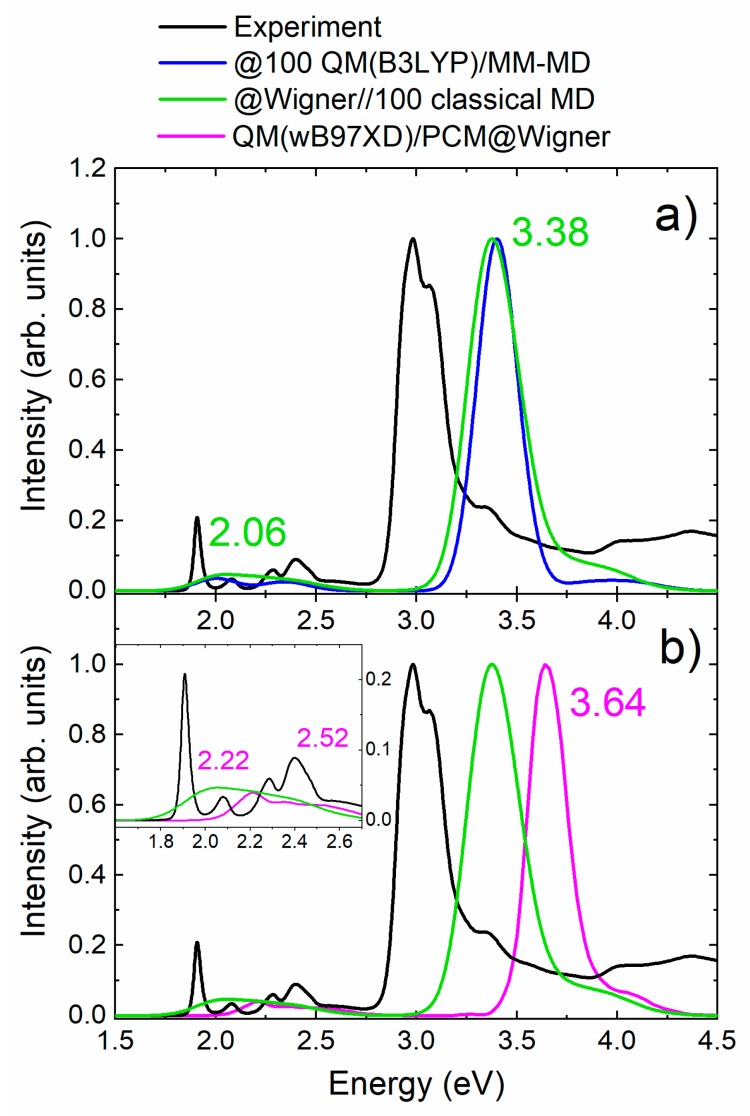
(**a**) Comparison of the hybrid-Wigner-MD spectrum and its energies at the maxima (green) with the @100 QM(B3LYP)/MM-MD (blue) and with the experimental absorption spectrum of mTHPC in methanol (black). (**b**) Comparison between QM(wB97XD)/PCM@Wigner (pink) and QM(wB97XD)/MM@hybrid-Wigner-MD (green) absorption spectra with respect to the experimental absorption spectrum of mTHPC (black) scaled with respect to the Soret band. The inset zooms the Q’ and Q′′ bands.

The so-obtained hybrid-Wigner-MD-based spectrum is shown in green in Figure 3a against the one previously discussed obtained from the short QM/MM-MD simulations (blue). As one can see, the differences are insignificant (below 0.06 eV)—a clear sign that the initial problem of the classical MD sampling was not the anharmonicity of the molecular motion of the PS but rather that the whole gradient of mTHPC was poorly approximated with the general Amber force field and a quantum mechanical method is more suitable.

So far, all the calculations included explicit methanol molecules in the sampling and the excited-state calculations. At this point one could argue whether the solvent must be described explicitly, or a simpler implicit model would be sufficient to reproduce the experimental absorption spectrum. We have therefore employed the same ensemble of geometries obtained from the Wigner distribution and computed the excited states using the polarizable-continuum model (PCM) to describe the solvent. The comparison between explicit and implicit solvation of mTHPC using a quantum sampling (QM(wB97XD)/MM@hybrid-Wigner-MD versus QM(wB97XD)/PCM@Wigner) is reported in Figure 3b. At first sight, it is clear that explicit solvent returns a better agreement with the experimental Soret band. In fact, the Soret band computed with implicit solvent presents the largest error (0.66 eV) with respect to the experiment, with a blue-shift of 0.26 eV with respect to the explicit-solvent computation. The Q’ band, shown in the inset of Figure 3b, is also blue-shifted (0.16 eV) with respect to the band obtained with the explicit solvation QM/MM model. The results of Figure 3 suggest that, for this particular chromophore, the solvent cannot be approximated with a continuum model but instead the methanol molecules should be included explicitly in the configurational sampling and in the excited-state calculations.

Finally, it is interesting to investigate the importance of performing a long sampling to capture the slow motions of the solvent. To this aim, we compare two procedures of the QM(B3LYP)/MM-MD configurational sampling. In the first procedure a single simulation of 5 ps starting from the last snapshot of the classical MD simulation is performed. Since the simulation time is very short, the solvent could possibly not have enough time to sample the relevant configurational space. The second procedure is the @100-QM/MM-MD sampling approach described above, where 100 simulations are run for 100 fs starting from 100 snapshots selected from the last nanosecond of the classical MD trajectory. Since the starting structures for the QM/MM-MD simulations are chosen from a relatively long simulation time (one nanosecond), this last approach is expected to provide a better sampling of the configurational space of the solvent. 

**Figure 4:**
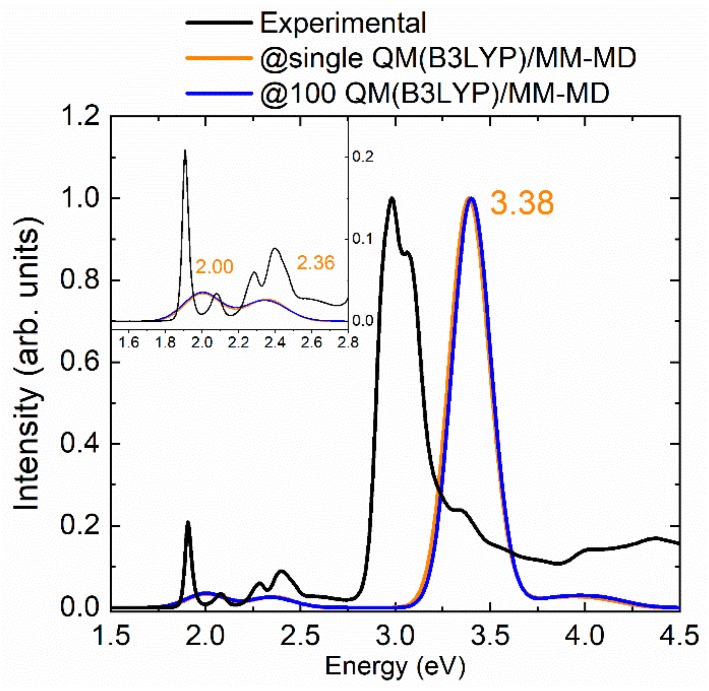
Comparison against the experimental reference (black) of the absorption spectra of mTHPC in methanol scaled with respect to the Soret band and computed from geometries obtained from @single-QM(B3LYP)/MM-MD simulation (orange) and from @100-QM(B3LYP)/MM-MD simulations (blue). The inset zooms the Q’ and Q’’ bands.

Figure 4 demonstrates that the differences between the spectra obtained with both approaches (single and multiple QM/MM-MD simulations) are negligible. The Q’ bands of the two sets of computations perfectly match, the Q′′ band is slightly blue-shifted by 0.02 eV in the case of the single QM/MM-MD simulation while the Soret band is red-shifted by the same amount. This result indicates that slow motions of the solvent are not involved in the electronic excitation process of mTHPC and, thus, the use of short MD simulations to sample the configurational space is adequate enough to obtain an accurate absorption spectrum. However, this conclusion should not be extrapolated to other solvated chromophores, for which a long sampling could be necessary.

### 2.2. Density of Triplet States

As mentioned before, mTHPC is a potent second-generation PS in photodynamic therapy. Cytotoxic species, such as singlet oxygen ^1^O_2_, fundamental for the therapeutic effect, are produced from the reaction of the PS in a triplet electronic excited state with compounds of the surrounding environment; this can be molecular oxygen or a more complex molecular substrate, such as the cell membrane. It is therefore interesting to compute the density of triplet states and to investigate how different computation protocols affect their energies.

**Figure 5:**
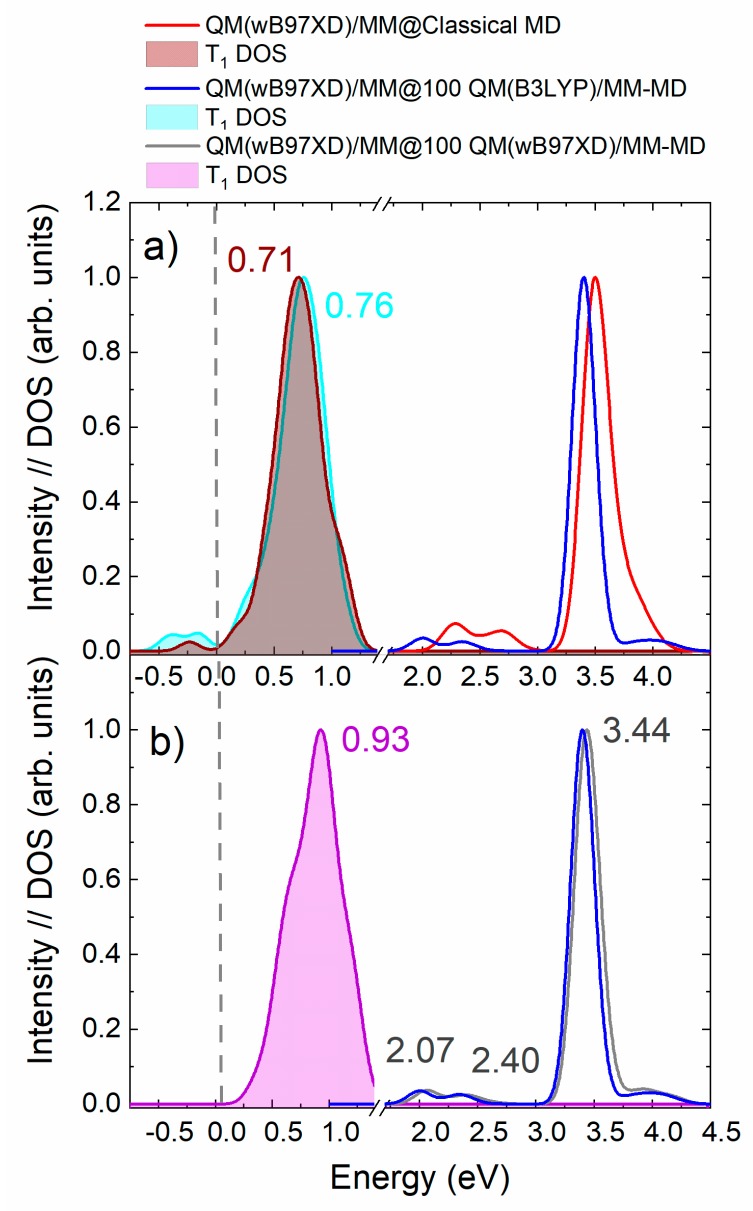
(**a**) Density of states (DOS) of the first triplet excited state T_1_ of mTHPC and the corresponding absorption spectrum obtained with the QM(wB97XD)/MM@Classical-MD (red curve for the absorption spectrum and wine for the DOS) and the QM(wB97XD)/MM@100-QM(B3LYP)/MM-MD approaches (blue curve for the absorption spectrum and cyan curve for the DOS). The centre of the T_1_ DOS is also reported in eV. (**b**) Absorption spectrum obtained with the QM/MM-MD sampling using B3LYP (blue) and wB97XD (grey) in the QM part. The DOS for the first triplet excited state calculated with the QM(wB97XD)/MM@100-QM(wB97XD)/MM-MD and the value at its centre is reported in purple.

To this aim, we have computed the density of states (DOS) of the lowest-lying triplet excited state of mTHPC using the QM(wB97XD)/MM@100-QM(B3LYP)/MM-MD protocol, which was the one providing the most accurate absorption energies. Figure 5a shows that the lowest-energy triplet-state T_1_ band presents a portion of its DOS close and below 0 eV. When the sampling is performed by classical MD, there is also a portion of the triplet band falling below zero. The problem of having negative triplet transition energies in TD-DFT calculations [36,48,50] can be partially ascribed to the exchange-correlation functional, where the amount of Hartree-Fock exchange [51] and the range-separated character [49] are the most delicate aspects in this matter. A second source of error in TD-DFT is the adiabatic approximation which assumes an instantaneous reaction of the exchange-correlation potential when the electron density changes in time. It has been shown that this approximation introduces large errors when computing spin-flip transitions, such as S_0_-T_1_ transitions, especially when range-separated functionals, for example, wB97XD, are used [49]. Exchange and correlational functionals that present relatively large contributions of the HF exchange have larger probability to display the so-called triplet instability problem, for which the triplet excitation energies approach the zero value [51].

Despite the above discussed possible reasons for the errors in the S_0_-T_1_ energies, we have found that the use of different functionals for sampling (B3LYP) and excited-state energies (wB97XD) also introduces a significant error. Indeed, if the same functional is used (wB97XD) for both types of calculations the negative energy problem vanishes, as can be seen in Figure 5b. The DOS of the first triplet excited state is now centred at 0.93 eV (i.e., blue-shifted by 0.17 eV with respect to using B3LYP) and there are no geometries with negative triplet states. Figure 5 also shows that the use of different functionals for sampling does not significantly affect the excitation energies of singlet excited states since the calculated absorption spectra based on QM(B3LYP)/MM-MD and QM(wB97XD)/MM-MD sampling protocols are similar, with a small blue shift of 0.07 eV of the latter with respect to the former. Therefore, the spectral bands computed with QM(wB97XD)/MM show a larger blue-shift for the triplets (0.17 eV) than for the singlets (0.07 eV) when the sampling is changed from QM(B3LYP)/MM-MD to QM(wB97XD)/MM-MD. In other words, the use of a different functional for sampling and excitation energies calculation introduces an artificial red-shift of the excitation energies, which is more important for the triplet states and thus causes the appearance of negative triplet-state energies for some geometries of the ensemble. 

This artificial red-shift in the excitation energies is also found in the static approach, reported in Table 1, where the excitation energies are computed only for the ground-state optimized geometry. Using B3LYP for the geometry optimization and wB97XD for the excited-state energy calculation within an implicit description of the solvent (PCM), we find that the S_n_ excitation energies are only shifted by a maximum of 0.21 eV to lower energies with respect to the values obtained using the wB97XD functional for both geometry optimization and excited-state calculations. Triplet electronic transitions T_n_, in contrast, suffer from stronger deviations and show red-shifts of even 0.45 eV when a different functional is adopted for the geometry optimization and the excited-state calculation.

It is also important to have in mind that the calculations that used the same functional for sampling and excitation energies, still employed different basis sets. Therefore, the level of theory is not completely identical. In particular, the basis set used for sampling was 6-31G*, whereas for the subsequent vertical excitations was 6-311G**. It is very likely that the use of different basis sets for sampling and excitation energies also introduces artificial shifts in the excitation energies. However, it is expected that these shifts are much smaller than the ones caused by the use of different functionals.

## 3. Conclusions

The absorption spectrum of mTHPC in methanol and its density of triplet states have been computed using an electrostatic-embedding QM/MM approach, in which the PS is treated with TD-DFT and the solvent with MM. Several ensembles of geometries produced by different sampling methods were employed with the aim to explore the impact of the way in which the configurational space of mTHPC in solution is obtained. The motivation behind the study was that the standard approach, where the system is sampled with a classical MD simulation, did not provide a satisfactory absorption spectrum (errors up to 0.29–0.52 eV) in comparison with the experimental absorption energies.

The predicted absorption energies improved (errors 0.42–0.06 eV) when mTHPC was described with a QM method (DFT-B3LYP/6-31G*) during the sampling step instead of MM, while the solvent molecules are still described with MM. Such improvement is due to the better description of the ground-state gradient of mTHPC by B3LYP than by the force field. Deficiencies due to anharmonicities in the normal modes of the molecule were excluded by doing a comparison of the spectra obtained with geometries extracted from QM/MM-MD simulations, which describe anharmonicities, and with geoemtries extracted from a Wigner distribution, which is fully harmonic. In addition, a long sampling of the solvent degrees of freedom is not needed to capture the most important solvent effects. It is important to highlight that the conclusions extracted for mTHPC in methanol should not be transferred to other systems where anharmonicities or large solvent rearrangements may play a crucial role. 

Since implicit models are often highly accurate and easily implemented in the excited-state calculations, we also questioned the suitability of implicit versus explicit solvent. In this case, we have employed the PCM model to describe the solvent as a continuum during the computation of the excitation energies. We found that the implicit solvation provided absorption energies that are in much worse agreement with the experiment (errors 0.66–0.22 eV).

Having found the best protocol to reproduce the experimental absorption spectrum of mTHPC in methanol (QM(wB97XD)/MM@100-QM(B3LYP)/MM-MD), we computed the density of triplet states. Interestingly, the use of two different functionals for the ground-state sampling and the subsequent excited states calculation led to triplet states having negative energies. This is because the potential-energy surfaces of triplet and singlet states suffer from different shifts when the functional is changed, resulting in a very similar absorption spectrum but rather different density of T_1_ states.

This work emphasizes that the sampling method employed to generate a configurational ensemble has a substantial impact in the calculation of electronically excited states. Snapshots that are refined with QM/MM-MD simulations, even if for a short time (here 100 fs), are highly desirable to improve the description of the geometrical parameters.

## 4. Materials and Methods

### 4.1. Computational Details

For the thermal sampling, the mTHPC molecular parameters were taken from the General Amber Force Field (GAFF) [52] with restrained electrostatic potential (RESP) charges computed by DFT, employing the B3LYP [53] functional and the 6-31G* basis set [54]. The drug molecule, placed in an octahedral box, was solvated with methanol up to 14 Å from any atom of the mTHPC molecule. The parameters of the solvent were taken from the Amber libraries [55]. 

The system was minimized for 10000 cycles, the first 5000 employing the steepest-descent algorithm whereas the last 5000 cycles were performed with the conjugate-gradient one. The minimized system was subsequently heated for 0.1 ns at 300 K in the canonical ensemble (NVT) followed by an equilibration step in the isothermal-isobaric (NPT) ensemble for 2 ns.

For all the simulations, periodic-boundary conditions were applied and a time step of 2 fs was employed. The SHAKE algorithm [56] was used to freeze bonds involving H atoms. The Langevin thermostat was used with a collision frequency gamma of 1.0 ps^−1^ while the pressure was kept fixed at 1 atm with the Berendsen barostat and isotropic position scaling. The cut-off for the non-bonded interactions was set to 10.0 Å and the particle-mesh Ewald method was used to calculate the coulombic interactions [57].

Snapshots from the classical MD simulation were used as starting structures for the QM/MM-MD simulations to refine the structure of mTHPC. The chromophore was included in the QM region, whereas the surrounding methanol molecules are in the region described classically. The interaction between both regions was computed using an electrostatic-embedding scheme. First, we have performed a simulation of 5 ps, starting from the last snapshot of the classical MD, describing mTHPC at the DFT level with the B3LYP [53] functional and the 6-31G* basis set [54]. This simulation is labelled as single-QM/MM-MD in Section 2. Then, from 100 snapshots taken from the last nanosecond of the classical MD simulation, we have restarted 100 short QM/MM-MD simulations which lasted from 75 fs up to 125 fs in order to randomize and increase the configurational sampling. This procedure was performed describing the QM region with DFT employing both B3LYP [53] and wB97XD [58] functionals and the 6-31G* basis set [54].

The same 100 snapshots from the classical MD simulation were also used to perform a hybrid sampling, where the geometries of mTHPC were replaced with the ones belonging to the B3LYP-gas phase-Wigner distribution. The geometry of the chromophore and the vibrational frequencies were computed in the gas phase at the DFT/B3LYP/6-311G** [53,54] level of theory. Such new system was then again equilibrated in the NPT ensemble for one additional ns to allow the solvent to adapt to the new structure of the chromophore, which was kept frozen with the ibelly option all along the simulation. The final snapshots of these 100 MD simulations formed the hybrid Wigner/classical-MD ensemble. Finally, the 100 geometries generated by the quantum sampling (B3LYP-gas-phase Wigner) were also used in excited-state QM/PCM calculations to compare implicit and explicit solvation models. We have used the linear-response DFT/PCM formulation and the IEFPCM formalisms of the PCM model. The cavity was built employing the atomic radii defined by the UFF force field. The static dielectric constant of the solvent (methanol) is ε = 32.613 and dielectric constant at optical frequency is ε_opt_ = 1.77.

The absorption spectra were obtained from the 6 lowest-energy excited states computed for the 100 geometries provided by the different configurational sampling methods described above using the wB97XD functional [58] and the 6-311G** basis set [54]. The lowest-energy triplet transition was also computed to get the corresponding density of triplet states. The spectra and density of states were obtained convoluting the excited states with gaussian functions with a full width at half maximum of 0.2 eV.

All the MD simulations were performed with the AMBER16 program [59] interfaced with Gaussian09 [60] for the QM/MM-MD simulations. The vertical excitations, DFT geometrical optimizations and frequency calculations were performed with Gaussian09 software [60].

### 4.2. Experimental Methods

UV/vis absorption spectra were recorded using a spectrophotometer Specord S600 from Analytik Jena (Jena, Germany) using 1x1cm Suprasil^®^ quartz cuvettes (HELLMA, Müllheim, Germany). Temoporfin (mTHPC) was taken from a lot delivered by biolitec pharma Ltd. (Dublin, Ireland). The methanol solvent used to prepare the Temoporfin solution was of spectroscopic grade (MERCK, Uvasol^®^). The obtained experimental spectrum of Temoporfin in methanol was normalized to 1 at the highest absorption peak in the UV/vis region and agrees with the peaks reported in Ref. [17,18,61].

## Figures and Tables

**Table 1:** Excitation energies in eV of the lower lying singlet and triplet states of mTHPC computed with the static approach at the QM(wB97XD)//QM(wB97XD) and the QM(wB97XD)//QM(B3LYP) level of theory. The energy differences between the two methods is also reported in eV.

	QM(wB97XD)//QM(wB97XD)	QM(wB97XD)//QM(B3LYP)	ΔE (eV)
S_1_	2.21	2.01	0.20
S_2_	2.60	2.39	0.21
S_3_	3.40	3.28	0.12
S_4_	3.50	3.36	0.14
S_5_	4.14	4.10	0.04
S_6_	4.36	4.27	0.09
T_1_	1.07	0.92	0.15
T_2_	1.61	1.16	0.45
T_3_	2.05	1.98	0.07
T_4_	2.44	2.33	0.11

## Supplementary Materials

The following are available online.
