# Supplementary material for: Assessing Configurational Sampling in the Quantum Mechanics/Molecular Mechanics Calculation of Temoporfin Absorption Spectrum and Triplet Density of States
Source: Molecules. 2018 Nov 9;23(11):2932. doi: 10.3390/molecules23112932 (PMC6278509; doi:10.3390/molecules23112932)
Supplement: Supplementary file 1 [file molecules-23-02932-s001.pdf]

SUPPORTING INFORMATION

Assessing configurational sampling in the QM/MM calculation of Temoporfin absorption spectrum and triplet density of states.

Martina De Vetta,[1,2] Omar Baig, [1] Dorika Steen, [3] Juan J. Nogueira,[1,4,*] and Leticia González[1,*]

1 Institute of Theoretical Chemistry, Faculty of Chemistry, University of Vienna, Währinger Str. 17, 1090, Vienna (Austria);
2 Departamento de Química Universidad Autónoma de Madrid Francisco Tomas y Valiente, 7, 28049 Cantoblanco, Madrid (Spain);
3 biolitec research GmbH, Otto-Schott-Str. 15, 07745 Jena (Germany);
4 Current address: Research School of Biology, Australian National University, Canberra, ACT 2601, Australia
* Correspondence: leticia.gonzalez@univie.ac.at, juanjose.nogueiraperez@anu.edu.au; Tel.: +43--1-4277-52750

S1 Benchmarking of Exchange-Correlation Functionals at the Franck-Condon Geometry

In Figure S1 we report the spectral lines corresponding to the most relevant features of the absorption spectrum of mTHPC in methanol, identified as Soret band and Q′ and Q′′ bands. The first six singlet excited states have been computed with time-dependent density-functional theory (TD-DFT) at the Franck-Condon (FC) geometry. The geometry was optimized using the same functional used for the vertical-energy calculations. We have adopted the xc-functionals reported in the legend of Figure S1 and the 6-311G** basis set [1] and including the solvent methanol by means of the implicit PCM solvation model [2].

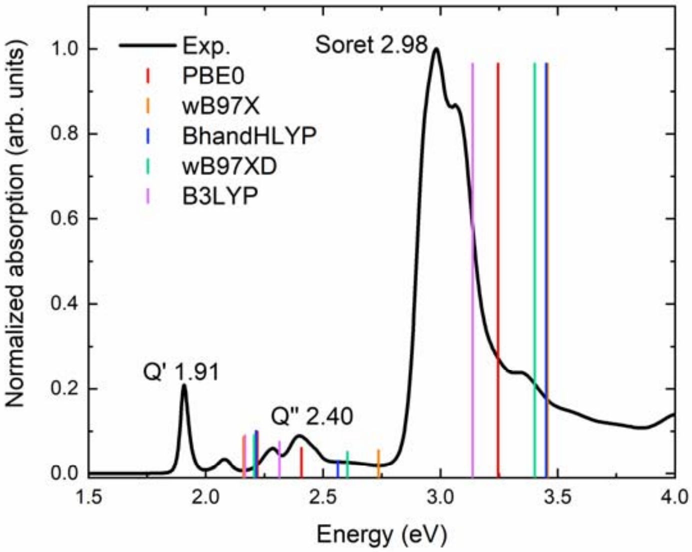

Figure S1. Spectral lines of the electronic transitions corresponding to the experimental Q′, Q′′ and Soret absorption bands computed with TD-DFT using the functionals listed in the legend.

The selection of the functionals for this study have been guided from existing literature on the computation of the absorption spectrum of mTHPC. For instance, the study of Eriksson and Eriksson [3], which considers the accuracy of long-range corrected functionals in the prediction of the absorption features

of tetrapyrrole derivatives. This work asserts wB97X as the most precise functional for the most red-shift absorption feature, which we have identified as Q′ band. A second study deals with the effect of the chlorin structure on the Q′ band [4]. It reports similar and tolerable errors for the other three functionals considered here, i.e., BhandHLYP, PBE0 and B3LYP, in comparison with other more expensive computational methods. The two standard hybrid functionals (PBE0 and B3LYP), having a 20-30% of exact exchange, are also the ones that generally provide small mean errors in TD-DFT benchmark studies [5–7].

Comparison of the selected functionals with the experimental absorption spectrum in the FC vertical approximation is reported in Figure S1 and Table S1. The errors in the prediction of the excitation energies of the most red-shifted Q-bands (Q′ and Q′′) and the most intense Soret band are different for all the functionals. The smallest error for the Q′ absorption band is given by wB97X, immediately followed by B3LYP. Regarding the errors in the computed absorption energies of the Q′′ band, the best functionals are PBE0 and B3LYP, whereas wB97X is the functional that gives the largest error (0.34 eV). Finally, for the most intense Soret band, the error is very large for wB97X, wB97XD and BhandHLYP (> 0.40 eV), while PBE0 and B3LYP predict more reasonable energies with the latter having the smallest error (0.16 eV). Therefore, considering the excitation energies at the optimized ground-state geometry corresponding to the three absorption bands, B3LYP provides the most accurate results.

Table S1. Experimental and theoretical excitation energies in eV for the most relevant absorption features of the mTHPC absorption spectrum. The error of the theoretical prediction with respect to the experimental value is also reported in eV.

	Experiment	PBE0	wB97X	BhandHLYP	wB97XD	B3LYP
**Q′ band**						
Energy (eV)	1.91	2.22	2.16	2.21	2.21	2.17
Error (eV)		0.31	0.25	0.31	0.30	0.26
**Q′′ band**						
Energy (eV)	2.40	2.41	2.74	2.56	2.60	2.31
Error (eV)		0.01	0.34	0.16	0.20	0.09
**Soret band**						
Energy (eV)	2.98	3.25	3.46	3.45	3.40	3.14
Error (eV)		0.26	0.47	0.47	0.42	0.16

S2 Benchmarking of Exchange-Correlation Functionals Considering an Ensemble of Geometries

We examine the performance of different functionals when vibrational sampling is introduced by means of classical molecular dynamics (MD) and the excitation energies for each geometry of the ensemble is computed by an electrostatic-embedding quantum mechanics/molecular mechanics (QM/MM) scheme, where the QM region is described by the different functionals. We find in Figure S2 two distinct groups of spectra according to the functional employed for the TD-DFT calculations. The first group is formed by the PBE0 and B3LYP spectra, which present the smallest error for the Soret band (0.21 and 0.09 eV for PBE0 and B3LYP, respectively). The second group, formed by the BhandHLYP, wB97X and wB97XD spectra, has larger errors for the Soret band. Among them, the spectrum computed with wB97XD is the one which presents the most accurate energy (3.50 eV) in comparison with the experimental band (2.98 eV). The related functional wB97X predicts the worst excitation energy and finds the Soret band at 3.62 eV. The BhandHLYP functional predicts

an intermediate value of 3.55 eV. All the tested functionals overestimate the absorption energy of the Soret band.

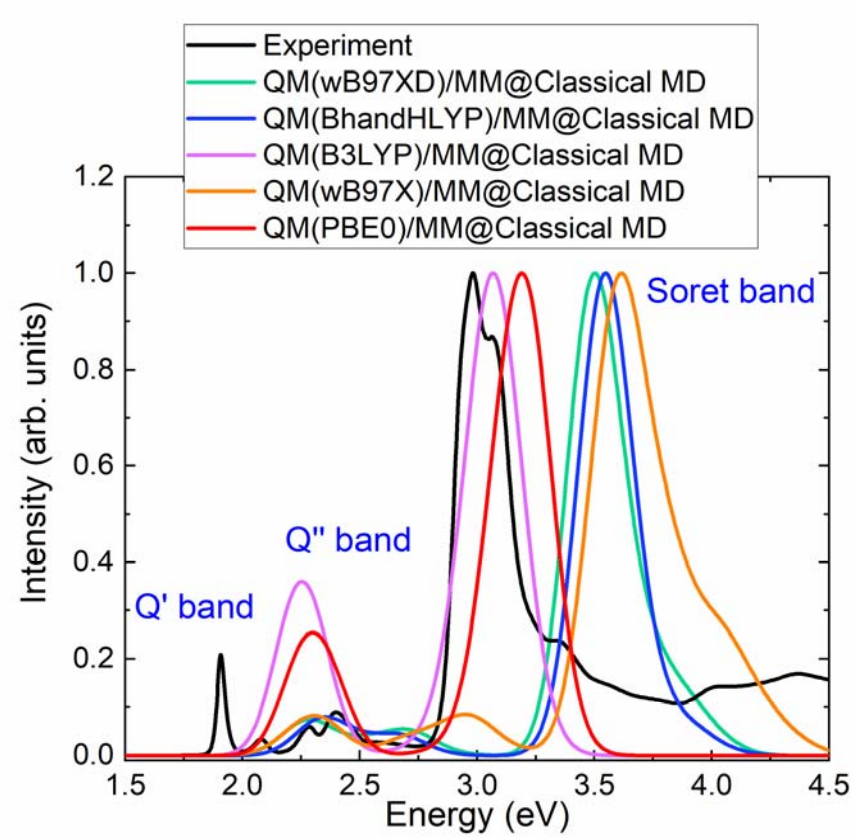

Figure S2. Absorption spectrum of mTHPC computed with the levels of theory reported in the legend and based on an ensemble of configurations provided by a classical MD simulation. The experimental spectrum is reported for comparison.

The two functionals that best predicted the Soret absorption energy (see Figure S2 and S3), are not able to reproduce the shape of the experimental spectrum in the lower energy region, where the Q′ and Q′′ bands are found. In fact, as reported in Figure S3, the B3LYP and PBE0 functionals predict a unique band centered at 2.25 and 2.30 eV, respectively. On the contrary, all the others functionals considered find two distinct absorption features at lower energies which can be related to the two experimental Q′ and Q′′ bands. Focusing on the absorption energy of the Q′ band, we see from Figure S3 that the two related functionals wB97X and wB97XD outperform BhandHLYP. Specifically, the Q′ band is predicted at 2.30 and 2.29 eV by the wB97X and wB97XD functionals, respectively, and at 2.35 eV by BhandHLYP.

The Q′′ band seems to be more difficult to estimate. First we notice that wB97X predicts this feature at rather high energies and with an erroneous similar absorption intensity to the Q′ band. A more realistic relative absorption intensity and absorption energy is instead predicted for the two others functionals wB97XD and BhandHLYP, which find this absorption band at 2.69 and 2.66 eV, respectively, and with smaller intensity than the Q′ band.

Therefore, among all the functionals considered we find wB97XD as the most suitable one to describe the absorption of mTHPC with QM/MM schemes, especially taking into account the low energy region of the

spectrum, which is the relevant one for PDT applications. This functional in fact not only reproduces well the energy of the two experimental absorption bands Q′ and Q′′, but it also correctly predicts the relative absorption intensities of the Q bands with respect to the Soret band and with respect one another. Finally, this is also the functional that also gives the most red-shift values for the excitation energy of the Q′ band.

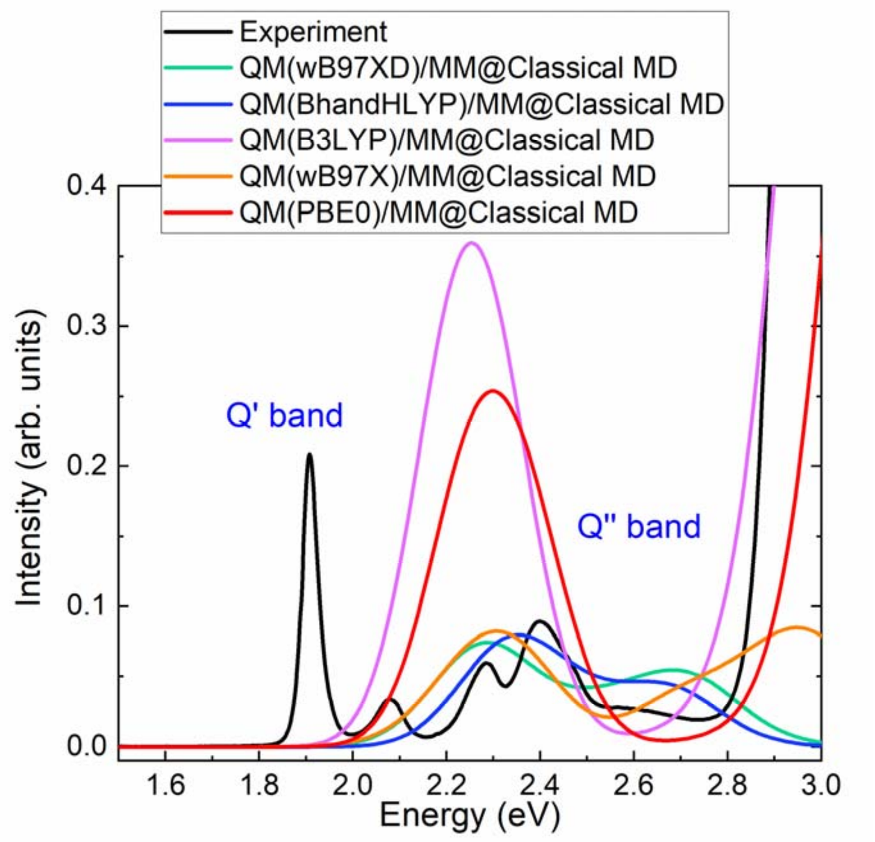

Figure S3. Focus on the most red-shift part of the spectra reported in Figure S2 where the absorption bands labelled as Q′ and Q′′ are found.

S3 Polarization Effects

Polarization of the solvent induced by the electronic excitation of the chromophore may cause modifications in the absorption spectrum. Since in the employed electrostatic-embedding QM/MM partition of the system all methanol molecules are described by a fixed-charged force field in the MM region, solvent polarization is not accounted in our calculations. One accurate way of considering solvent polarization is to include some of the solvent molecules in the QM region. In order to select the methanol molecules to be included in the QM region, we chose the solvent molecules that during the classical MD simulation are involved in hydrogen bonds with the mTHPC molecule for at least more than 20% of the total simulation time according to an hydrogen-bond analysis performed with cpptraj [8]. We consider that a hydrogen bond is formed when the separation between the hydrogen donor and the hydrogen acceptor atoms is smaller than 3.0 Å and the angle formed by these two atoms and the hydrogen atom is larger than 135°. We found that 12 methanol molecules are usually involved in hydrogen bonding and they were therefore included in the QM region during the

QM/MM computation of the spectrum. This spectrum was computed by using the same 100 geometries from the 100 short QM/MM-MD simulations that were employed in the calculations shown in Figures 2, 3 and 4. Since we are using TD-DFT for the excited states calculations, unrealistic charge-transfer (CT) states have to be handle with care. To assure that the spectrum does not contain unrealistic lower-lying CT states between the mTHPC molecule and the methanol molecules included in the QM region we have excluded these state by performing a quantitative analysis of the one-electron transition density with the TheoDORE wavefunction analysis software [11]. Specifically, first, we have subdivided the QM region of the system in two fragments, one containing the mTHPC molecule and the other containing the 12 QM solvent molecules. Then, the CT number [9,10], which provides the fraction of electron transferred between the two fragments, is computed. Among all excited states computed, none of them has a CT character superior to 0.1, therefore all the states can be considered local states of the chromophore and are considered when obtaining the spectrum.
The polarization effect expected from the inclusion of the most relevant methanol molecules when computing the absorption energies cannot really be appreciated only by looking at the absorption spectra reported in Figure S4. The differences between the QM/MM spectrum with 12 solvent molecules in the QM region and the one computed with the standard QM/MM scheme (with all solvent molecules in the MM region) are in fact not significant in this case. Therefore, one can conclude that, for this particular system, polarization solvent effects do not alter the shape of the absorption spectrum of the chromophore.

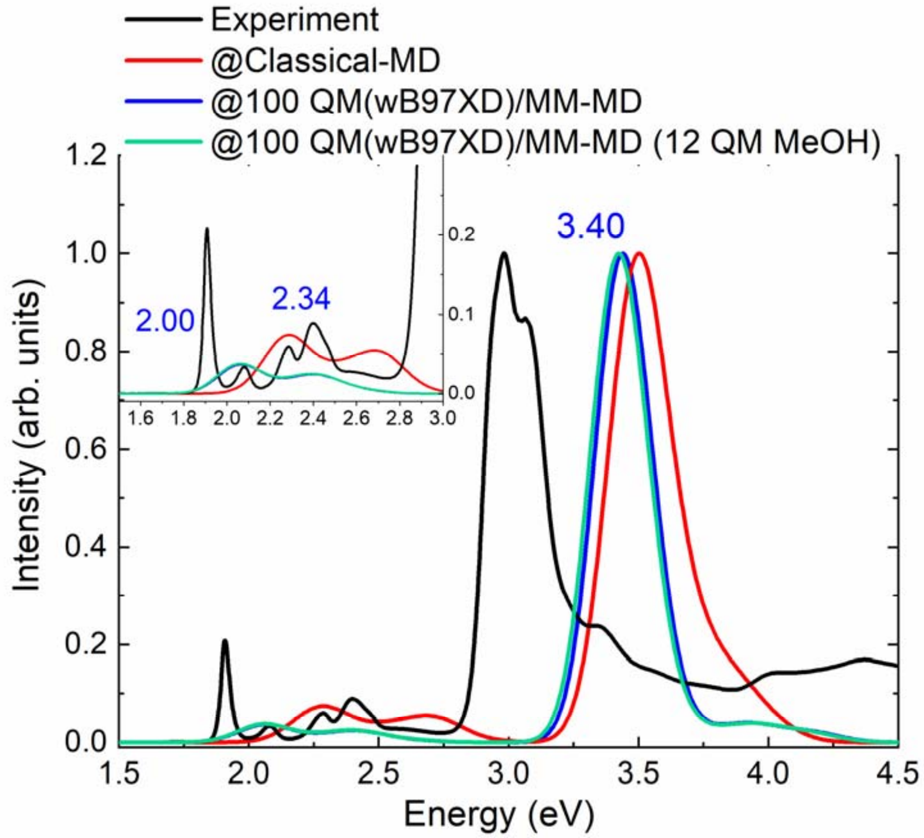

Figure S4. Comparison of the absorption spectrum computed from the classical MD ensemble of geometries and the one obtained after the QM/MM refinement. From the second ensemble the excited states have been computed considering all methanol molecules as MM region or including 12 molecules in the QM region.

REFERENCES

[1] P.J.A. Ditchfield R., Hehre, W. J., An Extended Gaussian-Type Basis for Molecular-Orbital Studies of Organic Molecules., J. Chem. Phys. 54 (1971) 724–728.

[2] B. Mennucci, Polarizable continuum model, Wiley Interdiscip. Rev. Comput. Mol. Sci. 2 (2012) 386–404. doi:10.1002/wcms.1086.

[3] E.S.E. Eriksson, L. a Eriksson, Predictive power of long-range corrected functionals on the spectroscopic properties of tetrapyrrole derivatives for photodynamic therapy., Phys. Chem. Chem. Phys. 13 (2011) 7207–17. doi:10.1039/c0cp02792h.

[4] M. Palma, G.I. Cardenas-Jiron, M.I.M. Rodriguez, Effect of chlorin structure on theoretical electronic absorption spectra and on the energy released by porphyrin-based photosensitizers, J. Phys. Chem. A. 112 (2008) 13574–13583. doi:10.1021/jp804350n.

[5] F. Santoro, D. Jacquemin, Going beyond the vertical approximation with time-dependent density functional theory, Wiley Interdiscip. Rev. Comput. Mol. Sci. 6 (2016) 460–486. doi:10.1002/wcms.1260.

[6] D. Jacquemin, A. Planchat, C. Adamo, B. Mennucci, TD-DFT assessment of functionals for optical 0-0 transitions in solvated dyes, J. Chem. Theory Comput. 8 (2012) 2359–2372. doi:10.1021/ct300326f.

[7] D. Jacquemin, B. Mennucci, C. Adamo, Excited-state calculations with TD-DFT: From benchmarks to simulations in complex environments, Phys. Chem. Chem. Phys. 13 (2011) 16987–16998. doi:10.1039/c1cp22144b.

[8] D.R. Roe, T.E. Cheatham, PTRAJ and CPPTRAJ: Software for processing and analysis of molecular dynamics trajectory data, J. Chem. Theory Comput. 9 (2013) 3084–3095. doi:10.1021/ct400341p.

[9] J.J. Nogueira, F. Plasser, L. González, Electronic delocalization, charge transfer and hypochromism in the UV absorption spectrum of polyadenine unravelled by multiscale computations and quantitative wavefunction analysis, Chem. Sci. 8 (2017) 5682–5691. doi:10.1039/c7sc01600j.

[10] F. Plasser, A.J.A. Aquino, W.L. Hase, H. Lischka, UV absorption spectrum of alternating DNA duplexes. Analysis of excitonic and charge transfer interactions, J. Phys. Chem. A. 116 (2012) 11151–11160. doi:10.1021/jp304725r.

[11] F. Plasser, S.A. Bäppler, M. Wormit, A. Dreuw, New tools for the systematic analysis and visualization of electronic excitations. II. Applications, J. Chem. Phys. 141 (2014) 24107. doi:10.1063/1.4885820.
